# Metastasectomy in Leiomyosarcoma: A Systematic Review and Pooled Survival Analysis

**DOI:** 10.3390/cancers14133055

**Published:** 2022-06-21

**Authors:** Megan Delisle, Bader Alshamsan, Kalki Nagaratnam, Denise Smith, Ying Wang, Amirrtha Srikanthan

**Affiliations:** 1 Division of General Surgery, The Ottawa Hospital, University of Ottawa, Ottawa, ON K1N 6N5, Canada; megandelisle@gmail.com; 2 Department of Medicine, College of Medicine, Qassim University, Buraydah 52571, Saudi Arabia; bshmsan@qu.edu.sa; 3 Department of Medicine, University of Ottawa Faculty of Medicine, Ottawa, ON K1N 6N5, Canada; 4 Interdisciplinary School of Health Sciences, Faculty of Health Sciences, University of Ottawa, Ottawa, ON K1N 6N5, Canada; knaga018@uottawa.ca; 5 Health Sciences Library, McMaster University, Hamilton, ON L8S 4L8, Canada; dsmith@mcmaster.ca; 6 Division of Medical Oncology, BC Cancer—Vancouver Cancer Centre, Vancouver, BC V5Z 4E6, Canada; ying.wang@bccancer.bc.ca; 7 Department of Medicine, University of British Columbia, Vancouver, BC V6T 1Z4, Canada; 8 Division of Medical Oncology, The Ottawa Hospital, Ottawa, ON K1H 8L6, Canada; 9 Ottawa Hospital Research Institute, Ottawa, ON K1H 8L6, Canada

**Keywords:** sarcoma, metastasis, leiomyosarcoma, metastasectomy, surgery, survival, systematic review

## Abstract

**Simple Summary:**

Leiomyosarcoma (LMS) is an aggressive soft tissue sarcoma with a poor prognosis. Approximately 40% of patients will develop metastatic disease. The optimal treatment for patients with metastatic LMS is not well established, and there are no randomized controlled trials regarding metastasectomy. This systematic review and pooled survival analysis aims to assess the survival in patients undergoing a metastasectomy for LMS and compare the outcomes based on the site of metastasectomy. We identified that patients with LMS metastases in the lungs, liver, spine, and brain can undergo metastasectomy with acceptable survival. Two studies have compared survival outcomes between patients treated and not treated with metastasectomy; despite their low quality, these studies support a survival benefit associated with metastasectomy.

**Abstract:**

This study assesses the survival in patients undergoing metastasectomy for leiomyosarcoma (LMS) and compares the outcomes by the site of metastasectomy. We conducted a systematic review and pooled survival analysis of patients undergoing metastasectomy for LMS. Survival was compared between sites of metastasectomy. We identified 23 studies including 573 patients undergoing metastasectomy for LMS. The pooled median survival was 59.6 months (95% CI 33.3 to 66.0). The pooled median survival was longest for lung metastasectomy (72.8 months 95% CI 63.0 to 82.5), followed by liver (34.8 months 95% CI 22.3 to 47.2), spine (14.1 months 95% CI 8.6 to 19.7), and brain (14 months 95% CI 6.7 to 21.3). Two studies compared the survival outcomes between patients who did, versus who did not undergo metastasectomy; both demonstrated a significantly improved survival with metastasectomy. We conclude that surgery is currently being utilized for LMS metastases to the lung, liver, spine, and brain with acceptable survival. Although low quality, comparative studies support a survival benefit with metastasectomy. In the absence of randomized studies, it is impossible to determine whether the survival benefit associated with metastasectomy is due to careful patient selection rather than a surgical advantage; limited data were included about patient selection.

## 1. Introduction

Leiomyosarcoma (LMS) is a malignant mesenchymal tumor arising from smooth muscle cells that accounts for 10–20% of soft tissue sarcomas [1,2]. LMSs most commonly occur in the uterus, followed by the abdomen, the retroperitoneum, and larger blood vessels [3]. LMSs are principally tumors of adults and are more common in women [3]. Most LMSs are sporadic, but some may be associated with hereditary syndromes, such as retinoblastoma and Li-Fraumeni. Compared to other histologic types of soft tissue sarcomas (STS), LMSs are inherently aggressive, with 90% of patients diagnosed with grade two or three tumors [4,5]. LMSs have a poorer prognosis with a tendency for distant recurrence and a decreased disease-free survival [6,7].

Surgery to achieve negative margins remains the only curative treatment modality for patients presenting with localized LMS. Adjunctive therapies, such as radiotherapy and systemic treatment, are used in only specific cases [8,9,10]. Despite optimal local treatment, the risk of developing metastatic disease is approximately 40% [11]. The optimal treatment for patients with metastatic LMS is not well established, and there are no randomized controlled trials regarding metastasectomy. Many studies on this topic include multiple sarcoma histologies, limiting generalizability to distinct individual histologies, which can vary in clinical course, outcome, and sensitivity to radiotherapy and systemic therapy. Most patients with metastatic LMSs are not curable, and palliative systemic or radiotherapy is the mainstay of management. Retrospective studies have demonstrated an association with improved survival in carefully selected patients. The role of metastasectomy is most well accepted for patients with oligometastatic pulmonary metastases, but other sites of metastasectomy are increasingly reported in the literature [12,13,14]. This study aims to assess the survival in patients undergoing metastasectomy for LMS and compare the outcomes based on the site of metastasectomy.

## 2. Materials and Methods

This study is a part of a series systematically summarizing survival outcomes for patients with soft tissue and bone sarcoma undergoing metastasectomy. This study focuses on survival outcomes of patients who underwent metastasectomy for LMS. Details on information sources, search strategy, eligibility criteria, study screening and selection, data collection, and extraction can be found elsewhere [15]. The protocol is registered within the prospective international register of systematic reviews (PROSPERO) database (registration ID: CRD42019126906), and this study is reported in compliance with PRISMA 2020 statement [16].

### 2.1. Search Strategy

The literature search was developed by a research librarian (D.S.). The search included Medline, Embase, Cochrane Central Register of Controlled Trials, and ClinicalTrials.gov from inception to 28 May 2021, and a PubMed search for studies not yet indexed or not found in Medline. The search strategy was tailored to each database. Conference abstracts for the last three years from three major sarcoma conferences were also searched: the Connective Tissue Oncology Society, the American Society of Clinical Oncology, and the European Society of Clinical Oncology. Reference lists of all included studies and relevant systematic reviews were reviewed for additional references.

### 2.2. Selection Process

We included studies that evaluated metastasectomy for LMS with survival outcomes, were peer-reviewed in the English language, and had a minimum of five patients with LMS undergoing metastasectomy. Studies that included a broad range of cancer histologies (sarcoma and non-sarcoma histologies) and reported the survival outcomes for the subgroup of patients undergoing metastasectomy for LMS were included. These studies did not have to report the sociodemographic and clinical data for the subgroup of LMS patients to be included. Four reviewers (working in pairs—B.A., M.D., A.S., and Y.W.) screened titles and abstracts independently and in duplicate in the first stage, then reviewed the full texts of potentially eligible studies in a second stage to determine the final eligible studies. Disagreements were resolved by referring to a third reviewer if necessary.

### 2.3. Data Collection

Data were extracted by two individual members (B.A. and M.D.) and compared for accuracy. A third member (A.S.) reviewed the data extraction and resolved inconsistencies where necessary. When patients undergoing metastasectomy for LMS were a subgroup of the entire study population, two attempts at contacting primary authors were made to obtain LMS-specific patient and treatment data. If still unavailable, these data were extracted for the entire study population.

### 2.4. Data Synthesis and Analysis

The details of the included articles are presented in table format. The LMS-specific baseline data were included when studies reported the sociodemographic and clinical characteristics of patients diagnosed with LMS undergoing metastasectomy [17,18,19,20,21,22,23,24]. Among studies with a broad range of cancer types, of which LMS was included, the sociodemographic and clinical characteristics of patients with LMS undergoing metastasectomy were not consistently reported [11,13,14,25,26,27,28,29,30,31,32,33,34,35,36]. Thus, these characteristics are reported for the entire study population to provide details despite representing multiple cancer histologies. The LMS-specific survival outcomes were reported by all studies and are summarized in table format.

The yearly Kaplan–Meier estimated survival rates and numbers at risk for LMS patients were extracted from each study. For studies where these data were not reported, if the Kaplan–Meier curves indicated the time at which patients were censored or a risk table was provided, this was used to derive the patient-level data from the study. For studies reporting Kaplan–Meier curves of overall survival, WebPlotDigitizer v4.5 was used to identify the follow-up time and estimated survival rate at each “step” of the curve [37]. If censoring times were not available, then *IPDfromKM* web-based Shiny application was utilized to reconstruct individual patient data from published Kaplan–Meier curves [38]. The numbers of deaths and numbers at risk at each year of the follow-up period were then used to calculate standard errors for the yearly survival estimates and median overall survival. If only median overall survival was reported and Kaplan–Meier curves or risk tables were not available, the standard error was calculated using methods described by Hozo et al. [39]. Median overall survival and yearly survival estimates were then pooled across studies using inverse-variance weighted random-effects meta-analysis models [40].

### 2.5. Risk of Bias Assessment and Certainty of Evidence

Risk of bias assessments were completed by two individual members (B.A. and K.N.), with a third member (M.D.) resolving disagreements where necessary. First, the study design was determined using accepted definitions [41]. Studies reporting survival for both metastasectomy and non-metastasectomy patients were defined as cohort studies. Studies reporting survival for only metastasectomy patients were defined as case series. Patients who did not undergo metastasectomy may have received other treatments, such as chemotherapy or radiation.

The Joanna Briggs Institute (JBI) Critical Appraisal Checklist for Case Series and the Newcastle-Ottawa Quality Assessment Scale (NOS) were selected as the methodological quality assessment tools based on expert recommendations [42,43,44]. Specific decision trees were developed and agreed upon by all authors to adjudicate each criterion.

The constructs of the GRADE (Grading of Recommendation, Assessment, Development, and Evaluation) approach to assess the certainty of evidence were applied [45]. Although we did not perform a comparative meta-analysis, the components of GRADE can still be used to address evidence synthesis of quantitative estimates of effect (and thus summarized narratively) [46].

## 3. Results

### 3.1. Study Characteristics

Out of 37,241 articles, 23 studies published between 1998 and 2020 were included (Supplementary Figure S1, Table 1) [11,13,14,17,18,19,20,21,22,23,24,25,26,27,28,29,30,31,32,33,34,35,36]. Twenty-one studies were case series, [13,14,17,18,19,20,21,22,23,24,25,26,27,28,29,30,31,32,33,34,35] and two were cohort studies [11,36]. Collectively, the articles included 1970 patients diagnosed between 1976 and 2018, of which 656 (33%) were diagnosed with metastatic LMS and 573 (29%) underwent metastasectomy for LMS (Supplementary Table S1).

Eight studies reported the sociodemographic and clinical data for patients diagnosed with LMS undergoing metastasectomy (Table 2A) [17,18,19,20,21,22,23,24]. The other 15 studies included a broad range of cancer types, of which metastatic LMS was a subgroup and the survival outcomes for patients undergoing metastasectomy for LMS were explicitly reported (Table 2B) [11,13,14,25,26,27,28,29,30,31,32,33,34,35,36]. The proportion of patients undergoing metastasectomy for LMS in these studies ranged from 8% [25] to 60% [34].

### 3.2. Sociodemographic and Clinical Characteristics of Patients Undergoing Metastasectomy for LMS

The sociodemographic and clinical data for patients with LMS undergoing metastasectomy were available for 113 patients from eight studies and will be discussed here (Table 2A) [17,18,19,20,21,22,23,24]. The mean or median age was between 47 and 58, with individual patient age ranges between 23 and 76. Fifty-eight (51%) patients were male. The most common site of origin of LMS was gastrointestinal (*n* = 34, 30%), uterine/adnexal (*n* = 33, 29%), retroperitoneal (*n* = 23, 20%), extremity/trunk (*n* = 17, 15%), other (*n* = 6, 5%), and vena cava (*n* = 1, 1%). The primary tumor in patients undergoing metastasectomy was reported to be well controlled (no additional details provided) in six studies [17,18,19,20,21,23].

Seven studies reported either the disease-free interval (DFI) or the proportion of patients presenting with synchronous versus metachronous metastatic disease [17,18,19,20,21,22,23]. Fourteen patients (23%) had synchronous disease and 47 (77%) had metachronous disease. The median DFI was between 15 and 50 months, with an individual patient range between zero and 204 months. The most common sites of metastases included liver (*n* = 59, 42%), lung (*n* = 47, 33%), spine (*n* = 18, 13%), peritoneum (*n* = 7, 5%), lymph nodes (*n* = 5, 4%), other (*n* = 4, 3%), bone (*n* = 1, 1%), and adrenal (*n* = 1, 1%).

### 3.3. Management of Patients Undergoing Metastasectomy for LMS

Out of 656 patients with metastatic LMS included in all 23 studies, 573 (87%) underwent at least one metastasectomy (Table 3). The most commonly reported site of metastasectomy for LMS was lung (*n* = 353, 62%) followed by liver (*n* = 165, 29%), spine (*n* = 39, 7%), and brain (*n* = 5, 1%). The site of metastasectomy was not specified for 11 (2%) patients. Nine studies reported the intent for metastasectomy, and the criteria used to select patients for metastasectomy were reported by ten studies (Table 4).

Six studies reported whether perioperative systemic therapy was used in patients undergoing metastasectomy for LMS, of which 48 (52%) received perioperative systemic treatment [11,17,18,19,20,24]. Only three studies reported the type of systemic therapy used [11,18,19]. Van Cann et al. reported that seven out of 28 patients received systemic treatment before their first metastasectomy, of which four received an anthracycline combined with an alkylating agent regimen, two received a single-agent anthracycline, and one received the oral tyrosine kinase inhibitor, pazopanib [11]. Chen et al. reported that four out of 11 patients received perioperative systemic therapy; one patient received adriamycin, dacarbazine, and etoposide preoperatively, and, postoperatively, one patient received doxorubicin, dacarbazine, ifosfamide, and mesna, another received doxorubicin, dacarbazine, and etoposide, and a third received cytoxan and vincristine [18]. Faraj et al. reported that two out of five patients with synchronous disease who underwent the simultaneous resection of all disease received postoperative chemotherapy [19]. One patient received doxorubicin and ifosfamide and another received doxorubicin alone [19].

Five studies reported whether perioperative radiotherapy was used in patients undergoing metastasectomy for LMS, of which 18 (20%) received perioperative radiotherapy [11,17,18,20,24]. The details of the radiotherapy’s type, dose, and frequency were not consistently reported.

### 3.4. Post-Metastasectomy Outcomes

For the assessment of overall survival, the median follow-up time ranged from 14 to 60 months across the studies (Supplementary Table S2). All 23 studies reported either a median overall survival or a one-year, three-year, or five-year overall survival for patients with LMS undergoing metastasectomy (Supplementary Table S2).

Kaplan–Meier curves or risk tables were available in 14 studies, allowing for individual patient data to be extracted and pooled yearly survival estimates to be calculated [13,17,18,19,20,21,22,23,24,25,28,29,34,36]. Two additional studies reported the median overall survival and range, from which the standard error could be calculated, and were included in the pooled median overall survival analysis [11,14].

**Figure 1:**
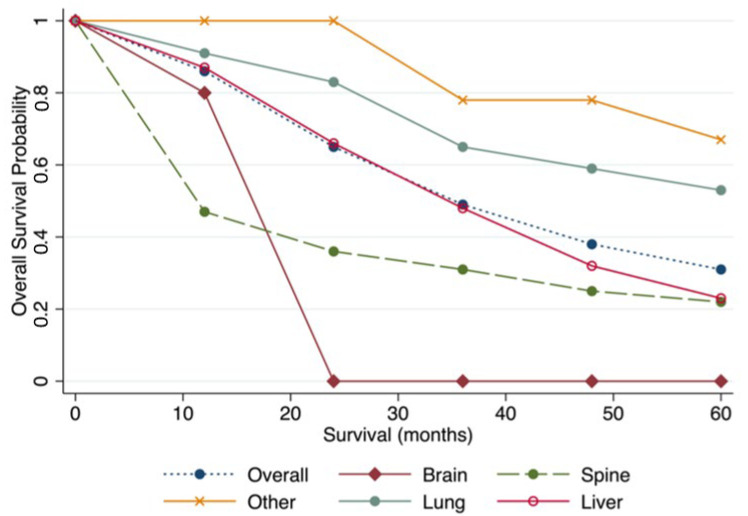
Pooled overall survival by site of metastasectomy.

The pooled median survival was 59.6 (95% CI 33.3 to 66.0) months. The pooled median overall survival was longest for patients undergoing lung metastasectomy (72.8 months 95% CI 63.0 to 82.5), followed by liver (34.8 months 95% CI 22.3 to 47.2), spine (14.1 months 95% CI 8.6 to 19.7), and brain (14 months 95% CI 6.7 to 21.3). The yearly pooled overall survival estimates are available in Table 5, and the yearly pooled estimates by the site of metastasectomy are displayed in Figure 1. Patients undergoing lung and liver metastasectomy did better than those undergoing brain and spine metastasectomy (Figure 1).

Two studies compared survival outcomes for patients with metastatic LMS versus those who did not undergo metastasectomy [11,36]. Both these studies reported metastasectomy was for curative intent; however, neither presented the criteria used to select patients for metastasectomy. Van Cann et al. found that among patients who underwent metastasectomy, the median overall survival was 83 months (range 4–127) compared to 16 months (range 0–83) among those who did not undergo metastasectomy (multivariable analysis HR 0.4 95% CI 0.2–0.8 *p* = 0.01) [11]. Farid et al. found that among patients who underwent metastasectomy, the median overall survival was 205 months (range 45–205) compared to 40 months (range 5–140) among those who did not [36]. On univariable analysis, those who did not undergo metastasectomy were at a significantly higher risk of death compared to those who did (HR 5.30 95% CI 1.52–18.49 *p* = 0.004), and this risk was even higher in a subgroup analysis of patients with lung metastases (HR 9.09 95% CI 1.16–100 *p* = 0.012) [36].

### 3.5. Prognostic Factors Associated with Post-Metastasectomy Outcomes

#### 3.5.1. Lung

Burt et al. identified that patients with a longer DFI had an improved overall survival on multivariable analysis (DFI included as a monthly continuous variable, HR 0.97 95% CI 0.94–0.99 *p* = 0.001) [17]. Paramanathan et al. identified that patients with a more favorable International Registry of Lung Metastases prognostic group (i.e., those with a completely resectable single metastasis with a DFI greater than 36 months) had improved survival (survival outcomes not reported quantitatively by authors) [47].

#### 3.5.2. Liver

Chen et al. identified that patients undergoing an R0 resection had a significantly longer median overall survival (median overall survival not reached, range 19–55 months) than those undergoing an R1/2 resection (median overall survival 25 months range 18–39 *p* = 0.03) [18]. Chen et al. also found no difference in survival between high- versus low-grade LMS, the number of liver metastases, the size of liver metastases, or the extent of liver resection [18]. Lang et al. found a prolonged survival among those undergoing first liver resections for metastatic disease who achieved an R0 resection (median overall survival 32 months range 1–84, five-year overall survival 20%) compared to an R1/2 resection (median overall survival 21 months range 1–49 *p* = 0.31, five-year overall survival 0%) [22]. Lang et al. also identified that patients undergoing liver resection for synchronous disease had a lower median overall survival than those with metachronous disease (22 versus 32 months, respectively, *p* = 0.61) [22]. Lang et al. did not find the presence of an extra-hepatic tumor to be associated with worse survival if they were able to achieve an R0 resection [22].

#### 3.5.3. Spine

Kato et al. assessed for various prognostic factors in univariable analyses and found postoperative Eastern Cooperative Oncology Group (ECOG) status was the only significant predictor of three-year overall survival after spine metastasectomy [20]. The three-year overall survival of patients with a postoperative ECOG status greater than three was 0% compared to 78% among those with an ECOG less than three (*p* = 0.003) [20].

### 3.6. Recurrence Post-Metastasectomy

Six studies reported recurrence post-metastasectomy for patients with LMS [17,19,20,21,23,24]. Of those, including patients who underwent lung metastasectomy, Burt et al. identified that 25 out of 31 patients recurred, of which 11 were managed with repeat metastasectomy [17]. Paramanthan et al. reported that eight out of 13 developed a recurrence [23]. Only one underwent repeat metastasectomy [23]. Of patients undergoing liver metastasectomy for LMS, Faraj et al. reported that all patients included in their study died of metastatic disease; the site of recurrence and management of recurrence was not specified [19]. Kim et al. reported that five out of 10 patients developed a recurrence. Two of these patients were managed with additional surgery. Among patients who underwent spine metastasectomy, Kato et al. reported that all patients included in their study died of metastatic disease, but the site of recurrence and the management of recurrence was not specified [20]. Ziewacz et al. reported that five out of eight patients recurred in their spine, of which, four underwent additional surgery and experienced improvement in their symptoms [24].

The outcomes of patients undergoing repeat metastasectomy were only reported by Lang et al.; the five-year overall survival was 0% and the median overall survival was 31 months (range 5–51) among the nine patients undergoing a second and third liver metastasectomy [22].

### 3.7. Risk of Bias and Certainty of Evidence

The risk of bias assessments are available in the supplementary material (Supplementary Tables S3 and S4). All included studies were at risk of bias. Based on the risk of bias assessments and review of the studies, the certainty of the bias was deemed very low (Supplementary Table S5).

## 4. Discussion

The role of metastasectomy in LMS is not currently well described in the literature. This study is the first to systematically synthesize and critique the available literature on this topic, thereby providing specific data that clinicians can generalize to LMS patients with metastases. We identified only two studies comparing the survival outcomes between patients who did, versus who did not undergo metastasectomy, which suggested an improved survival associated with surgery. In the absence of randomized studies, it is impossible to determine whether these findings are due to careful patient selection and favorable biology rather than a surgical advantage, as limited data was included in the publications about patient selection. However, most metastatic LMS are caused by high-grade tumors that are not indolent in their clinical behavior, and patients with metastatic LMS often have a poor prognosis without treatment.

Among patients undergoing metastasectomy for LMS, we found a pooled five-year overall survival of 31% (95% CI 18–44%) and a median overall survival of 59.6 months (95% CI 33.3 to 66.0). Before our study, the survival outcomes of patients undergoing metastasectomy for LMS were derived from large retrospective cohort studies with diverse histologies and were mostly limited to lung metastasectomy [27,48,49]. In these studies, the five-year overall survival post-lung metastasectomy ranged between 34 and 40%, with a median overall survival of 33 months. Compared to other histologic types of STSs, lung metastasectomy for LMS is suggested to be associated with a more favorable prognosis, and our results confirm this [27]. We estimated the pooled five-year overall survival among patients undergoing lung metastasectomy was 53% (95% CI 39–67%) and the median overall was 72.8 months (95% CI 63.0 to 82.5). Considerably less evidence exists describing the outcomes of patients undergoing metastasectomy for LMS at other sites. Our results suggest that patients with liver metastasectomy may also experience acceptable survival post-metastasectomy. In contrast, spine and brain metastasectomy may be more appropriately considered in palliative situations to improve quality of life.

We aimed to identify criteria that could be used to guide clinicians in the selection of patients with LMS appropriate for metastasectomy. The criteria used to select patients and the intent of metastasectomy were not uniformly reported by all studies. It was often not detailed enough to be used or replicated in clinical practice when reported. For example, the authors most commonly described selecting patients for metastasectomy if they had a long DFI, limited sites of metastatic disease, and demonstrated disease stability on chemotherapy. Additional considerations were noted to guide the selection of patients undergoing spine and brain metastasectomy, including their estimated prognosis, current performance status, and symptom burden. However, the specific details of how these criteria were evaluated or defined were not available, limiting the ability of clinicians to use these meaningfully in their clinical practice.

We identified that some patients undergoing liver (13, 34%), spine (1, 10%), and brain (2, 40%) metastasectomy had synchronous disease compared to none undergoing lung metastasectomy. In addition, patients undergoing liver (DFI range 16–50 months) and brain (DFI range 9–89 months) metastasectomy had a shorter median DFI compared to those undergoing lung (DFI range 26–48 months) and spine (DFI range 32–50 months) metastasectomies. Patients with brain and spine metastases are more prone to experience symptoms that impair their quality of life and could be eased by metastasectomy. For these reasons, patients with unfavorable prognostic characteristics, such as a short DFI and a synchronous presentation, may be more likely to be evaluated for metastasectomy if the treatment can improve their quality of life. However, it is unclear why there are more patients with synchronous disease and a shorter DFI undergoing liver compared to lung metastasectomies. It may be to decrease the systemic tumor burden, which may be associated with improved survival when resection of the primary tumor site is also performed. This difference in patient characteristics for those undergoing liver versus lung metastasectomy may partly explain why patients with lung metastasectomy had the most prolonged survival on pooled analysis. Developing more rigorous criteria for selecting patients who can benefit from metastasectomy is a priority for future research.

We found that few prognostic factors were evaluated quantitatively. Metachronous disease, a longer DFI, and R0 metastasectomy were favorable prognostic factors among lung and liver metastasectomy patients. The study by Paramanathan et al. was the only one to define a long DFI (i.e., 36 months) based on the International Registry of Lung Metastases prognostic group [23]. Patients undergoing lung metastasectomy were less likely to have additional sites of metastases compared to those undergoing liver metastasectomy. Interestingly, patients undergoing liver metastasectomy with extrahepatic disease who achieved complete resection of all disease had comparable survival to those without extrahepatic disease. This is an important finding, as patients with multiple sites of metastatic disease are often less likely to be considered for metastasectomy. For patients undergoing spine metastasectomy, post-metastasectomy performance status was the only significant prognostic factor. This has limited clinical utility as it is often difficult to predict how patients will respond to surgery. Additional research is required to determine which patients should be selected and who are most likely to benefit from metastasectomy.

We found that perioperative systemic and radiotherapy were infrequently utilized among patients undergoing metastasectomy for LMS. There is currently no evidence to support these treatment modalities in the perioperative metastatic setting. On the other hand, in the context of unresectable, metastatic STS, there is evidence to support cytotoxic chemotherapy. Anthracyclines, with or without ifosfamide, are regarded as an acceptable first-line treatment in this setting [50,51,52,53]. Many of the patients included in this systematic review were treated when our understanding of the various histologic types of STS was limited and before the practice of histology-driven treatment [10,53,54]. LMS has moderate sensitivity to ifosfamide-based regimens. As single therapies, doxorubicin and ifosfamide have demonstrated response rates of between 10% and 25% in LMS [10]. Dacarbazine had an overall response rate of 16% as a single agent, and retrospective data indicate overall response rates of nearly 37% when used in combination with doxorubicin [55,56]. In addition, gemcitabine and docetaxel also have demonstrated activity in LMS and this combination is used as a first-line therapy in the metastatic setting in some jurisdictions [57,58]. Newer treatments, including trabectedin, pazopanib and eribulin, have shown promising results in metastatic, unresectable LMS in later line settings [59,60,61,62,63,64,65,66,67,68,69,70,71]. It is imperative to evaluate the role of metastasectomy in the era of these modern systemic therapy regimens, even for all STS. Furthermore, because the majority of patients undergoing metastasectomy for LMS experience disease recurrence within a short interval, it is imperative to apply new treatment modalities for these metastases.

There is increasing evidence to support the feasibility and effectiveness of local interventional treatments, such as radiofrequency ablation, cryoablation, and stereotactic body radiation therapy [72,73,74,75,76]. Hepatic artery embolization with or without chemotherapy and radioembolization are further interventional treatments for liver metastases that can now be used in conjunction with other treatments. None of the studies included in this systematic review compared these local treatments to metastasectomy. As with many other rare diseases, retrospective data constitute the strongest available evidence, and decision-making around the management of these complex patients should be based on patient preferences in the context of multidisciplinary management.

Despite the promising survival outcomes, our results show that patients undergoing metastasectomy for LMS experienced high recurrence rates. For example, the five-year disease-free survival of patients undergoing lung metastasectomy was 9%, and the median disease-free survival was reported to be between 6 and 40 months. The five-year disease-free survival of patients undergoing liver metastasectomy was 22%, with a median disease-free survival between 13 and 16 months. The disease-free survival was not reported for patients undergoing spine and brain metastasectomies. Some patients who experienced recurrences underwent additional metastasectomies; this was performed for patients with lung, liver, and spine metastases. Currently, repeat metastasectomy is most well described and accepted for patients with lung metastases from various STS histologies, with the median overall survival after repeat metastasectomy reported to range between 25 and 65 months [77,78,79,80]. Prognostic factors associated with an improved median overall survival after repeat lung metastasectomy in these studies include achieving R0 margins, low-grade tumors, one or two sites of metastatic nodules, and the largest size of metastases less than 2 cm. Our results suggest that repeat liver metastasectomy results in comparable survival to repeat lung metastasectomy, and repeat spine metastasectomy may be warranted to improve symptoms [22]. Additional information on the criteria used to select patients for repeat metastasectomy and more data on survival outcomes are required to understand the feasibility.

### Limitations

Limitations of the evidence in this review include the retrospective nature of the existing case series and cohort studies. These non-randomized studies introduce potential biases due to careful patient selection. Most of the survival outcomes reported were not stratified or adjusted based on important prognostic factors. Given the small sample size of many included, it is unlikely such a stratified analysis would have been possible. Being limited to small study samples also increases the risk of the “small-study effects,” where smaller studies are more likely to be published if they report larger or more significant effects [81]. This is particularly important if unadjusted or unstratified estimates are reported. Another important limitation is that some studies included patients before the widespread use of the c-kit receptor for differentiation of gastrointestinal stromal tumors (GIST) versus LMS, which can otherwise have similarities on histopathology [82,83]. This is important as the outcomes for patients with GISTs are much better compared to LMS, which may have biased the results, particularly for the cohort of LMS arising from the gastrointestinal tract undergoing liver metastasectomy, as this is commonly the presentation of GISTs [84].

## 5. Conclusions

Surgery is currently being utilized to manage LMS metastases to the lung, liver, spine, and brain. Although low quality, comparative studies support a survival benefit, but patient selection and tumor biology are likely to have influenced these results. Recommendations regarding which patients should be considered for metastasectomy are limited by the variability in the criteria used to select patients for metastasectomy across studies and the sites of metastases. The majority of patients undergoing metastasectomy experience disease recurrence within a short interval. Additional research is required to establish the role of metastasectomy in the era of modern systemic therapy regimens and local ablative techniques. Leveraging international collaborations and registry data is one way to move forward with more robust and nuanced patient assessments in this rare disease [85].

## Figures and Tables

**Table 1:** Study details.

Study	Country	Center(s)/Registry	Inclusion Dates	Study Design	Inclusion Criteria
Anraku, 2004	Japan	Metastatic lung tumor study group of Japan	1984–2002	Case series	Pulmonary metastasectomy for uterine malignancies
Blackmon, 2009	USA	University of Texas M. D. Anderson Cancer Center	1998–2006	Case series	Pulmonary metastasectomy for STS and bone sarcoma
Burt, 2011	USA	The Brigham and Women’s Hospital	1989–2004	Case series	Pulmonary metastasectomy for STS and bone sarcoma
Chen, 1998	USA	The Johns Hopkins Hospital	1984–1995	Case series	Hepatic metastasectomy for LMS
Chudgar, 2017	USA	Memorial Sloan Kettering Cancer Center	1991–2014	Case series	Pulmonary metastasectomy for STS
Deguchi, 2020	Japan	Six institutes in Japan	2002–2018	Case series	Brain metastasectomy for STS and bone sarcoma
Ercolani, 2005	Italy	University of Bologna	1990–2003	Case series	Hepatic metastasectomy for noncolorectal nonneuroendocrine tumors
Faraj, 2015	Lebanon	American University of Beirut Medical Center	1998–2009	Case series	Hepatic metastasectomy for colorectal LMS
Farid, 2013	Singapore	National University of Singapore	2002–2010	Cohort study	All LMS
Goumard, 2018	USA	University of Texas M. D. Anderson Cancer Center	1998–2015	Case series	Hepatic metastasectomy for non-GIST sarcoma
Kato, 2020	Japan	Kanazawa University	2005–2016	Case series	Spine metastasectomy for LMS
Kim, 2017	Korea	Asian Medical Center	2003–2015	Case series	Hepatic metastasectomy for intra-abdominal LMS
Lang, 2000	Germany	Hanover Medical School	1982–1996	Case series	Hepatic metastasectomy for LMS
Liebl, 2007	Germany	University Medical Centre	1990–2005	Case series	Pulmonary metastasectomy for STS
Lin, 2015	USA	University of California Los Angeles Medical Center	1990–2010	Case series	Pulmonary metastasectomy for STS and bone sarcoma
Marudanayagam, 2010	UK	Queen Elizabeth University Hospital	1997–2009	Case series	Hepatic metastasectomy for STS
Paramanathan, 2013	Australia	Peter MacCallum Cancer Center and St. Vincent’s Health	2001–2011	Case Series	Pulmonary metastasectomy for sarcoma of gynecologic origin and STS
Rao, 2008	USA	University of Texas M. D. Anderson Cancer Center	1993–2005	Case series	Spine resection for primary or metastatic STS or bone sarcoma
Smith, 2009	USA	Roswell Park Cancer Institute	1976–2000	Case series	Pulmonary metastasectomy for STS surviving longer than five years
Van Cann, 2018	Belgium	University Hospitals Leuven	2000–2014	Cohort study	Metastatic LMS
Zacherl, 2011	Austria	Medical University of Vienna and Medical University of Graz	1987–2006	Case series	Hepatic metastasectomy for STS
Zhang, 2015	China	Central Hospital of PLA	2000–2009	Case series	Hepatic metastasectomy for extremity STS surviving longer than five years
Ziewacz, 2012	USA	University of Michigan	2005–2011	Case series	Spine metastasectomy for LMS

**Table 2:** Sociodemographic and clinical characteristics of included patients from studies reporting (A) and not reporting (B) these details for patients with LMS undergoing metastasectomy.

**A.** **Sociodemographic and Clinical Characteristics of Patients from Studies Reporting These Details for the LMS Patients Undergoing Metastasectomy**									
**Study**		**Total # Undergoing Metastasectomy for LMS**	**Median Age Years (Range)**	**Male #**	**Primary Site Location #**	**Synchronous #/Metachronous #**		**DFI (Months) from Primary Tumor to Metastases**	**Site of Metastases #,^a^**
Burt, 2011		31	Mean 52 (SD ± 9.3)	7	Uterus 13; extremity 10; retroperitoneum 4; trunk 2; other 2	NR		Mean 48 (SD ± 61)	Lung 31
Chen, 1998		11	57 (30–69)	2	Retroperitoneum 5; gastric 3; small intestine 2; uterine/adnexal 1	NR		Mean 16 (SD ± 4, range 0–40 months)	Liver 11
Faraj, 2015		5	47 (24–69)	2	Colon 4; rectum 1	3/2		NR	Liver 5; adrenal 1
Kato, 2020		10	Mean 53 (24–69)	5	Retroperitoneum 3; uterus 2; stomach 2; extremity 2; maxillary sinus 1	1/9		Mean 50 (range 10–204)	Spine 10; liver 1; lymph nodes 1 peritoneum 3; lung 3
Kim, 2017		10	48 (38–69)	3	Retroperitoneum 5; pancreas 1; small bowel 2; colon 1; stomach 1	2/8		Median 15 (range 5–38)	Liver 10
Lang, 2000 ^b^		26	Mean 54 (23–67)	18	Stomach 8; small bowel 4; vena cava 1; kidney 1; colon 1; upper abdomen/stomach 5; retroperitoneum 5; not specified 1	8/15 ^c^		Median 33 (range 0–164)	Liver 23; peritoneum 4; bone 1; lymph nodes 4
Paramanathan, 2013 ^d^		12	58 (44–76)	0	Uterus 12; broad ligament/adnexal 1	0/13		Median 26 (range 7–156)	Lung 13
Ziewacz, 2012		8	Mean 51 (25–66)	3	Uterus 4; chest wall 1; extremity 2; retroperitoneum 1	NR		NR	Spine 8
**B.** **Sociodemographic and Clinical Characteristics of Metastatic Patients of Studies Not Reporting These Details for the LMS Patients Undergoing Metastasectomy,^e^**									
**Study**	**Total # Included**	**Total # Undergoing Metastasectomy for LMS**	**Median Age Years (Range)**	**Male #**	**Histology #**	**Primary Site Location #**	**Synchronous #/Metachronous #**	**DFI (Months) from Primary Tumor to Metastases**	**Site of Metastases #,^a^**
Anraku, 2004	133	11	Mean 56 (26–80)	0	Squamous cell carcinoma 58; adenocarcinoma 13; endometrial adenocarcinoma 23; choriocarcinoma 16; LMS 11; other 12	Uterine 133	8/125	Range 0–243 months (0 months 8; 1–11 months 23; 12–35 months 38; ≥36 months 60)	Lung 133; extra-pulmonary 8
Blackmon, 2009	234	41	Mean 43 (8–83)	123	Osteosarcoma 46; MFH 33; SS 29; LMS 41; other 85	Extremity 136; NR 98	NR	NR	Lung only 147; lung + extra-pulmonary metastases 87
Chudgar, 2017	539	169	54 (15–90)	227	LMS 169; pleomorphic sarcoma/MFH 130; SS 81; other 81; fibrosarcoma 33; LPS 30; MPNST 15	Extremity 249; trunk 65; retroperitoneum/abdomen/pelvis 65; Visceral/GU/gynecologic 136; head and neck 24	71/468	Median 16 months (IQR 8–36)	Lung only 492; lung + extra-pulmonary metastases 47
Deguchi, 2020	22	5	45 (18–76)	11	ASPS 6; RMS 1; LMS 5, MPNST 1; osteosarcoma 1; epithelioid cell tumor 1; pleomorphic sarcoma 2 SS 2; undifferentiated sarcoma 1; UPS 2	NR	2/20	Median 20 months (range 0–267)	Brain 22; lung 19
Ercolani, 2005	83	10	Mean 55 (18–76)	35	NR	GI 18; breast 21; GU 15; soft tissue 10; other 19	11/72	≤1 year 34; >1 year 49	Liver 83
Farid, 2013 ^f,g^	97	11	51 (28–87)	23	LMS 97	Uterine 51; extremity 16; retroperitoneum 9; pelvis 8; GI 6; GU 5; other 2	27/NR	NR	Uterine LMS ^h^: liver 12.5%; lungs 81.3%; brain 6.3%; bones 12.5%; peritoneal 15.6%; lymph nodes 15.6%; others 25% Extrauterine LMS ^h^: liver 38.5%; lungs 50%; bones 11.5%; peritoneal 19.2%; lymph nodes 19.2%; others 26.9%
Goumard, 2018	126	62	54 (4–79)	56	LMS 62; LPS 14; hemangiopericytoma/SFT 9; vascular 7 (hemangioendothelioma 4; angiosarcoma 3); osteosarcoma 2; RMS 1; unclassified 26; NR 4	Abdominal 105; extra-abdominal 21	44/82	Median 12 months (range 0–298); >24 months 45	Liver 126; extra-hepatic metastases 26
Liebl, 2007	42	13	Mean 50 (17–73)	25	Alveolar sarcoma 2; extraskeletal chondrosarcoma 4; fibrosarcoma 2; LMS 13; MPNST 3; MFH 7; SS 4; spindle cell sarcoma 2; other 5	NR	10/32	Median 12 months; >18 months 16; ≤18 months 26	Lung 42
Lin, 2015	155	26	Mean 47 (11–92)	87	LMS 26; osteosarcoma 21; SS 19; chondrosarcoma 14; LPS 10; undifferentiated sarcoma/MFH 7; Ewing’s sarcoma 5; MPNST 5; alveolar soft part sarcoma 3; RMS 2; other 25; NR 18	Extremity 87; non-extremity 52; Visceral-gynecologic 16	23/132	Median 20 months (range 1–268)	Lung 155
Marudanayagam, 2010	36	20	58 (23–81)	13	Spindle cell sarcoma 1; angiosarcoma 1; osteosarcoma 1; carcinosarcoma 2; LPS 2; sarcomatoid renal cell tumor 4; GIST 5; LMS 20	Lung 1; vena cava 2; retroperitoneum 2; leg 3; skin 1; breast 1; ovary 1; uterus 3; kidney 4; colon 1; small bowel 5; mesentery 6; stomach 6	13/23	Median 17 months (range 0–322)	Liver 36; extra-hepatic metastases 11
Rao, 2008	80	21	53 (9–77)	NR	Chondrosarcoma 21; LMS 22; Osteosarcoma 10; LPS 9; RMS 1; SS 4; unclassified sarcoma 9; other 4	NR 51	NR/NR	Median 32 months (range 0–127)	Spine 51; active extraspinal disease 35
Smith, 2009	94	22	49 (9–75)	47	MFH 16; SS 18; LMS 22; LPS 12; other 26	Extremity 47; retroperitoneum 6; uterus 12; other 29	18/76	Median 15 months (range 0–176)	Lung 94; extra-pulmonary metastases 34
Van Cann, 2018 ^c^	122	28	60 (19–84)	45	LMS 122	Extremity 43; uterine 24; abdominal 23; vascular 13; GI 12; thoracic 5; cutaneous 2	38/84	Median 14 months (range 1–140)	Lung 78; liver 33; bone 9; lung only 47; liver only 10; bone only 3
Zacherl, 2011	15	9	Mean 62 (SD ± 12)	5	Pleiomorphic sarcoma 1; LMS 9; chondrosarcoma 1; GIST 2; malignant schwannoma 1; malignant GI autonomic nerve tumor 1	Small intestine 4; bone 3; pancreas 1; stomach 1; kidney 1; uterus 1; retroperitoneum 1; unknown primary 3	5/10	Median 33 months (range 15–124)	Liver 15
Zhang, 2015	27	12	42 (16–64)	15	LMS 12; SS 4; LPS 5; MFH 3; spindle cell sarcoma 3	Extremity 27	3/24	Median 31 months (range 0–104)	Liver 27

^a^ Patients may be included more than once; ^b^ Data for patients undergoing first liver metastasectomy; ^c^ Data only available for 23 patients; ^d^ One patient with endometrial stromal sarcoma included in the data presented; ^e^ Sociodemographic and clinical data listed in this table are for the entire metastatic cohort and includes patients diagnosed with LMS and other cancer histologies; ^f^ The entire study cohort included LMS patients of which only a subgroup underwent metastasectomy; ^g^ Sociodemographic and clinical characteristics reported are for both metastatic and non-metastatic patients at the time of diagnosis of the primary tumor; ^h^ Sites of metastatic disease were only reported as percentages stratified by uterine versus extrauterine sites of primary tumor. These include both synchronous and metachronous metastatic disease; NR: Not reported; #: Number of patients.

**Table 3:** Management of metastatic disease in studies reporting (A) and not reporting (B) these details for the LMS patients undergoing metastasectomy.

**A.** **Management of Metastatic Disease in Studies Reporting These Details for the LMS Patients Undergoing Metastasectomy**							
**Study**	**Site of Metastasectomy #,^a^**	**Number of Resected Metastases #**	**Size of Resected Metastases**	**Completeness of Metastasectomy #**	**Type of Resection #**	**Perioperative Systemic Therapy #**	**Perioperative Radiotherapy #**
Burt, 2011	Lung 31	Mean 1.9 +/− 1.5 (range 1–8)	Size of largest resected metastases 2.9 cm ± 2.4	R0 28; R1 3	Wedge 22; segmentectomy 2; lobectomy 7	Perioperative chemotherapy 20	Perioperative 7
Chen, 1998	Liver 11	Mean 2.6 (range 1–6)	Size of largest lesion mean 3.8 cm (range 1.1–10)	R0 6; R1/2 5	Segmentectomy 5; lobectomy 4; complex resection 2	Preoperative chemotherapy 1; postoperative chemotherapy 3	Preoperative 1
Faraj, 2015	Liver 5; adrenal 1	Multiple 5	Sze of largest metastases median 12 cm (range 6–16)	R0 3; unknown 2	Major hepatectomy 4; left adrenalectomy + right hepatectomy 1	Postoperative chemotherapy 2	NR
Kato, 2020	Spine 10	Solitary 10	NR	NR	Single vertebral resection 5; two or three consecutive vertebral resections 5	Preoperative chemotherapy 2; postoperative chemotherapy 6	Preoperative 2; postoperative 1
Kim, 2017	Liver 10	Solitary 6; multiple 4	Maximum size of metastasis median 2.6 cm (range 0.9–3)	R0 9; R1 1	Wedge 8; sectionectomy 1; right hepatectomy 1	NR	NR
Lang, 2000 ^b^	Liver 23	Solitary 10; two metastases 3; three metastases 4; >three metastases 6	Largest tumor diameter median 8 cm (range 2–25 cm)	R0 15; R1 3; R2 5	Segmentectomies 12, major hepatectomies 7, extracorporeal resections 4	NR	NR
Paramanathan, 2013	Lung 13	One metastasis 6; > one metastasis 7	NR	R0 11; R1 1; unresectable at the time of surgery 1	Wedge 7; segmentectomy 1; lobectomy 5;	Some patients had pre or postoperative chemotherapy ^c^	NR
Ziewacz, 2012	Spine 8	NR	NR	NR	Intralesional 8	Perioperative chemotherapy 7	Perioperative 6
**B.** **Management of Metastatic Disease in the Studies Not Reporting These Details for the LMS Patients Undergoing Metastasectomy, ^d^**							
**Study**	**Site of Metastasectomy #,^e^**	**Number of Resected Metastases**	**Size of Resected Metastases**	**Completeness of Metastasectomy #**	**Type of Resection**	**Perioperative Systemic Therapy #**	**Perioperative Radiotherapy #,^e^**
Anraku, 2004	Lung 133	One metastasis resected 77; 2–3 metastases resected 31; ≥4 metastases resected 23; NR 2	<3 cm 71; ≥3 cm 52; NR 10	NR	Pneumonectomy 3; bilobectomy 3; lobectomy 61 ^f^; wedge or segmentectomy 84 ^f^ Lung resection combined with mediastinal or hilar lymphadenectomy 45	NR	NR
Blackmon, 2009	Lung 234; abdomen 12; bone 16; brain 7; extra-pulmonary thoracic 3; pelvis 3; retroperitoneum 2; soft tissue/skin 7; scalp 5; spine 8	≤ Two 94; >2 132	NR	R0 184; R1 21; R2 29	For the first pulmonary resection only: Wedge 200; lobectomy, bilobectomy or sleeve 18; segmentectomy 15; pneumonectomy 1; Lung resection combined with lymph node dissection 7	NR	NR
Chudgar, 2017	Lung 539	One metastasis 229; 2 metastases 87; three metastases 57; four metastases 28; ≥5 metastases 138	NR	R0 490; R1 18; R2 31	Wedge 422; lobectomy 107; pneumonectomy 10	Preoperative chemotherapy 160; postoperative chemotherapy 53	NR
Deguchi, 2020	Brain 22	Single brain metastases 14; multiple brain metastases 8	Maximum metastasis size median 39 mm (range 5–80)	GTR 21; STR 1	NR	Postoperative chemotherapy 3; Postoperative tyrosine kinase inhibitor 3	WBRT 10; Stereotactic 12
Ercolani, 2005	Liver 83	Single metastases 58; multiple metastases 25	<5 cm 50; >5 cm 33	NR	Wedge resection 11; major hepatectomy 72	Postoperative chemotherapy 26	NR
Farid, 2013	NR	NR	NR	NR	NR	NR	NR
Goumard, 2018	Liver 126; resection of all extra-hepatic metastases 17	≥2 51	Maximum metastasis size 38 mm (range 3–330)	R0 107	Major liver resection 68; associated RFA 17; associated abdominal extrahepatic resection 37; associated thoracic extrahepatic resection 9	Preoperative chemotherapy 65; postoperative chemotherapy 33	Postoperative radiation 2
Liebl, 2007	Lung 42	Solitary 16; multiple 26	≤2 cm 22; >2 cm 20	NR	NR	Preoperative chemotherapy 12	NR
Lin, 2015	Lung 155	Average 4 +/− 4; range 1–29	Diameter of largest metastasis mean 2.9 cm +/− 3.0 (range 0.3–16)	R0 105; R1 13; R2 12; NR 25	Wedge 102; segmentectomy 20; lobectomy 27; pneumonectomy 6	Preoperative therapy not otherwise specified 93	
Marudanayagam, 2010	Liver 36; extra-hepatic metastases 11	Median 1 (range 1–6)	Maximum diameter of metastasis 11 cm (range 1–26)	NR	Segmentectomy 6; wedge 8; hemihepatectomy 17; trisectionectomy 5	NR	NR
Rao, 2008	Spine 51	NR	NR	NR	En bloc resection 6; intralesional resection 45	NR	NR
Smith, 2009	Lung 94; extra-pulmonary metastases 34	One pulmonary metastasis 34; >1 pulmonary metastasis 60	NR	R0 74; R1/2 20	Wedge resection 74; lobectomy 17; pneumonectomy 3	Postoperative chemotherapy 53	Perioperative radiation 7; intraoperative radiation 7
Van Cann, 2017	Lung 28	NR	NR	NR	NR	Perioperative systemic therapy 7	Postoperative radiotherapy 1
Zacherl, 2011	Liver 15	Solitary 5; multiple 10	Median tumor diameter 60 mm (range 20–200)	R0 10; R1 3; R2 2	Hemihepatectomy 9; Segmentectomy 4; wedge 3	Postoperative chemotherapy 4	NR
Zhang, 2015	Liver 27	<Two metastases 16; ≥2 metastases 11 Median 3 (range 1–13)	NR	R0 21; R1 6	Wedge 17; segmentectomy 8; Hemihepatectomy 2	Postoperative chemotherapy 22	NR

^a^ Patients may be included more than once; ^b^ Data presented for patients undergoing first metastasectomy only; ^c^ The number of patients that preoperative and postoperative chemotherapy was not reported^; d^ The management listed in this table are for the entire metastatic cohort and includes patients diagnosed with LMS and other types of cancers; ^e^ Patients may be included more than once; ^f^ Includes second resection of staged operation; NR: Not reported; R0: negative margins; R1: microscopically positive margin; R2: macroscopically/gross positive margin. NR: Not reported; #: Number of patients.

**Table 4:** Intent and criteria for metastasectomy reported by studies.

Study	Intent	Criteria
Anraku, 2004	NR	NR
Blackmon, 2009	Curative and palliative	Local control of the primary tumor. Immediate metastasectomy was recommended if there was a single or limited number of pulmonary metastases and a long DFI (minimum duration not specified) otherwise chemotherapy was recommended followed by metastasectomy if there was stable, responding, or slowly progressing disease.
Burt, 2011	Curative	Control of all extra-thoracic disease and lack of a better alternative systemic therapy.
Chen, 1998	NR	NR
Chudgar, 2017	NR	NR
Deguchi, 2020	Palliative	NR
Ercolani, 2005	Curative	Metastatic disease limited to the liver.
Faraj, 2015	Curative	NR
Farid, 2013	NR	NR
Goumard, 2018	NR	NR
Kato, 2020	NR	Solitary metastasis of the spine involving three or fewer consecutive spinal levels, an Eastern Cooperative Oncology Group Performance Status (ECOG) equal to or less than three, stable disease, and three or fewer metastases in other organs.
Kim, 2017	NR	NR
Lang, 2000	NR	NR
Liebl, 2007	NR	NR
Lin, 2015	NR	Chemotherapy followed by metastasectomy was preferred in patients with a short disease-free interval, multiple lesions involving both lungs, high-grade sarcoma, or when preoperative chemotherapy was recommended for the primary tumor in synchronous disease.
Marudanayagam, 2010	NR	Resectable with enough functional liver remanent, extrahepatic metastases a preclusion to hepatic resection.
Paramanathan, 2013	Curative	Control of the primary tumor and no extra-thoracic disease.
Rao, 2008	NR	NR
Smith, 2009	Curative	NR
Van Cann, 2018	Curative	NR
Zacherl, 2011	NR	Resectable with enough functional liver remanent.
Zhang, 2015	Curative	Metastatic disease limited to the liver.
Ziewacz, 2012	Palliative	Life expectancy of at least three years and neurological deficits, refractory pain, radiographic instability, or tumor progression despite chemotherapy and radiation.

NR: Not reported.

**Table 5:** Pooled overall survival estimates.

			1-Year Overall Survival		2-Year Overall Survival		3-Year Overall Survival		4-Year Overall Survival		5-Year Overall Survival	
Study	Site of Metastasectomy	Total #	# At Risk	Rate (%)	# At Risk	Rate (%)	# At Risk	Rate (%)	# At Risk	Rate (%)	# At Risk	Rate (%)
Anraku, 2003	Lung	11	7	64	5	55	4	38	3	38	2	38
Burt, 2011	Lung	31	29	98	25	87	19	72	16	64	13	52
Chen, 1998	Liver	11	11	100	7	72	4	52	1	35	0	0
Deguchi, 2020	Brain	5	2	80	0	0	0	0	0	0	0	0
Ercolani, 2005	Liver	10	8	80	6	60	6	60	5	50	3	30
Faraj, 2015	Liver	5	3	60	2	40	1	20	0	0	0	0
Farid, 2013	Other	11	11	100	9	100	7	78	7	78	6	67
Goumard, 2018	Liver	55	52	98	36	89	26	69	19	58	17	52
Kato, 2020	Spine	10	9	90	7	70	6	60	5	50	4	40
Kim, 2017	Liver	10	8	100	2	58	2	58	1	58	1	58
Lang, 2000	Liver	23	17	74	13	57	8	35	4	17	3	13
Paramanathan, 2013	Lung	13	12	92	11	92	8	76	6	66	4	66
Zacherl, 2011	Liver	9	5	56	5	56	3	33	1	11	1	11
Ziewacz, 2012	Spine	8	3	57	0	0	0	0	0	0	0	0
**Pooled overall survival (95% CI)**				**86 (78–94)**		**65 (52–79)**		**49 (36–62)**		**38 (24–53)**		**31 (18–44)**

#: Number of patients.

## Data Availability

Data sharing is not applicable to this article as no new data were created or analyzed in this study.

## Supplementary Materials

The following supporting information can be downloaded at: https://www.mdpi.com/article/10.3390/cancers14133055/s1, Figure S1: PRISMA Flow Diagram, Table S1: Total Patients Included in Each Study, Table S2: Post-Metastasectomy LMS-Specific Outcomes, Table S3: JBI Critical Appraisal Checklist for Case Series, Table S4: NOS for Cohort Studies, Table S5: Components of GRADE (Grading of Recommendation, Assessment, Development and Evaluation) to Assess the Certainty of Evidence.

## Abbreviations

ASPS	Alveolar soft part sarcoma
CI	Confidence interval
CSS	Cancer specific survival
DFI	Disease free interval
ECOG	Eastern Cooperative Oncology Group
GI	Gastrointestinal
GIST	Gastrointestinal stromal tumor
GTR	Gross total removal
GU	Genitourinary
IQR	Interquartile range
JBI	Joanna Briggs Institute
LMS	Leiomyosarcoma
LPS	Liposarcoma
MFH	Malignant fibrous histiocytoma
MPNST	Malignant peripheral nerve sheath tumor
NOS	Newcastle-Ottawa Quality Assessment Scale
NR	Not reported
OS	Overall survival
RMS	Rhabdomyosarcoma
SD	Standard deviation
SFT	Solitary fibrous tumor
SS	Synovial sarcoma
STR	Subtotal removal
STS	Soft tissue sarcoma
UPS	Undifferentiated pleomorphic sarcoma
WBRT	Whole brain radiation therapy
