# Supplementary material for: Metastasectomy in Leiomyosarcoma: A Systematic Review and Pooled Survival Analysis
Source: Cancers (Basel). 2022 Jun 21;14(13):3055. doi: 10.3390/cancers14133055 (PMC9264936; doi:10.3390/cancers14133055)
Supplement: Supplementary file 1 [file cancers-14-03055-s001.zip › Supplementary Table S1.pdf]

Table S1. Total Patients Included in Each Study

	Total Patients Included in the Study					
Study	Total included	Total with metastatic cancer	Total undergoing metastasectomy	Total with LMS	Total with metastatic LMS	Total undergoing metastasectomy for LMS
Anraku 2004	133	133	133	11	11	11
Blackmon 2009	234	234	234	41	41	41
Burt 2011	82	82	82	31	31	31
Chen 1998	11	11	11	11	11	11
Chudgar 2017	539	539	539	169	169	169
Deguchi 2020	22	22	22	5	5	5
Ercolani 2005	83	83	83	10	10	10
Faraj 2015	5	5	5	5	5	5
Farid 2013	97	NR	11	97	NR	11
Goumard 2018	126	126	126	62	62	62
Kato 2020	10	10	10	10	10	10
Kim 2017	10	10	10	10	10	10
Lang 2000	26	26	26	26	26	26
Liebl 2007	42	42	42	13	13	13
Lin 2015	155	155	155	26	26	26
Marudanayagam 2010	36	36	36	20	20	20
Paramanathan 2013	13	13	12	12	12	12
Rao 2008	80	51	51	21	21	21
Smith 2009	94	94	94	22	22	22
van Cann 2018	122	122	28	122	122	28
Zacherl 2011	15	15	15	9	9	9
Zhang 2015	27	27	27	12	12	12
Ziewacz 2012	8	8	8	8	8	8
Total patients	1970	1844	1760	753	656	573

NR: Not recorded
