# Supplementary material for: Metastasectomy in Leiomyosarcoma: A Systematic Review and Pooled Survival Analysis
Source: Cancers (Basel). 2022 Jun 21;14(13):3055. doi: 10.3390/cancers14133055 (PMC9264936; doi:10.3390/cancers14133055)
Supplement: Supplementary file 1 [file cancers-14-03055-s001.zip › Supplementary Table S2.pdf]

Table S2. Post-Metastasectomy LMS-Specific Outcomes

Study	Primary site of metastasectomy	Follow up duration post-metastasectomy (months)	Median OS months (range)	OS % (95% CI)	Median DFS months (range)	DFS % (95% CI)
Anraku 2004	Lung	Median 40 (range 0-220)	33 (1-100)	5-yr 38 (6-70)	NR	NR
Blackmon 2009	Lung	Median 27 (SD ± 33)	42 (NR)	NR	NR	NR
Burt 2011	Lung	NR	70 (0-100)	5-yr 52 (NR)	NR	NR
Chen 1998	Liver	Median 53 (range 23-98)	39 (18-55)	NR	NR	NR
Chudgar 2017	Lung	Median 27 (range 0-244)	42 (NR)	NR	6 (NR)	NR
Deguchi 2020	Brain	NR	14 (3-21)	NR	NR	NR
Ercolani 2005	Liver	Median 36 (range 6-168)	44 (13-87)	3-yr 64 (NR); 5-yr 37 (NR)	NR	3-yr 44 (NR); 5-yr 22 (NR)
Faraj 2015	Liver	NR	18 (7-135)	NR	NR	NR
Farid 2013	NR	NR	205 (45-205)	NR	NR	NR
Goumard 2018	Liver	Median 38 (range NR)	62 (10-120)	NR	13 (0-83)	NR
Kato 2020	Spine	Mean 47 (range 12-97)	45 (12-97)	3-yr 70 (NR)[a]; 5-yr 47 (NR)[a]	NR	NR
Kim 2017	Liver	NR	19 (8-170)	NR	NR	NR
Lang 2000[b]	Liver	NR	32 (1-84)	5-yr 13 (NR)	NR	NR
Liebl 2007	Lung	Median 27 (range NR)	27 (NR)	NR	NR	NR

Lin 2015	Lung	Mean 14 (range 1-192)	NR	3-yr 69 (NR); 5-yr 61 (NR)	NR	1-yr 49 (NR); 3-yr 9 (NR)
Marudanayagam 2010	Liver	Median 24 (range NR)	NR	5-yr 25 (NR)	NR	NR
Paramanathan 2013[c]	Lung	Median 26 (range 1-109)	76 (12-108)	3-yr 76 (NR); 5-yr 66 (NR)	40 (NR)[d]	NR
Rao 2008	Spine	Median 26 (range 1-131)	14 (9-19)	NR	NR	NR
Smith 2009	Lung	Minimum 60 for all patients	24 (NR)	NR	NR	NR
Van Cann 2018	Lung	Median 45 (range 19-127)	83 (4-127)	NR	NR	NR
Zacherl 2011	Liver	NR	32 (0-80)	NR	16 (0-64)	NR
Zhang 2015	Liver	NR	NR	5-yr 35 (NR)	NR	NR
Ziewacz 2012	Spine	NR	11 (3-23)	NR	NR	NR

[a] Cancer-specific survival; [b] First hepatic metastasectomy cohort; [c] One patient with endometrial stromal sarcoma included in the LMS patient data presented; [d] Uncontrollable disease-free survival (UDFS) was defined by the authors as being measured from the date of the first surgery to inoperability based on disease outside the chest, innumerable lung metastases, or exceeding the limit of respiratory reserve for further resections
