# Supplementary material for: Metastasectomy in Leiomyosarcoma: A Systematic Review and Pooled Survival Analysis
Source: Cancers (Basel). 2022 Jun 21;14(13):3055. doi: 10.3390/cancers14133055 (PMC9264936; doi:10.3390/cancers14133055)
Supplement: Supplementary file 1 [file cancers-14-03055-s001.zip › Supplementary Table S3.pdf]

Table S3. JBI Critical Appraisal Checklist for Case Series

	Question									
Study	1	2	3	4	5	6	7	8	9	10
Anraku 2004	No	Unclear	Unclear	Yes	Yes	No	No	No	Yes	Yes
Blackmon 2009	Yes	Yes	Yes	Yes	Yes	No	No	Yes	Yes	Yes
Burt 2011	Yes	Unclear	Unclear	Yes	Yes	Yes	No	No	Yes	Yes
Chen 1998	Yes	Unclear	Unclear	Yes	Yes	Yes	No	Yes	Yes	Yes
Chudgar 2017	Yes	Unclear	Unclear	Yes	Yes	No	No	Yes	Yes	Yes
Deguchi 2020	Yes	Yes	Unclear	No	Yes	No	Yes	No	Yes	Yes
Ercolani 2005	Yes	Unclear	Yes	No	Yes	No	No	No	Yes	Yes
Faraj 2015	Yes	Yes	Yes	No	Yes	Yes	No	Yes	Yes	Yes
Goumard 2018	Yes	Yes	Yes	Yes	Yes	No	No	Yes	Yes	No
Kato 2020	Yes	Yes	Yes	Yes	Yes	Yes	Yes	Yes	Yes	Yes
Kim 2017	Yes	Yes	Yes	Yes	Yes	Yes	Yes	No	Yes	No
Lang 2000	Yes	Unclear	Unclear	No	Yes	Yes	No	Yes	No	Yes
Liebl 2007	Yes	Unclear	Yes	Yes	Yes	No	No	Unclear	Yes	No
Lin 2015	Yes	Unclear	Unclear	Yes	Yes	No	No	Yes	Yes	Yes
Marudanayagam 2010	Yes	Unclear	Yes	Yes	Yes	No	No	Yes	No	No
Paramanathan 2013	Yes	Yes	Unclear	Yes	Yes	Yes	No	Unclear	Yes	Yes
Rao 2008	Yes	Unclear	Unclear	Yes	Yes	No	No	Yes	Yes	Yes
Smith 2009	Yes	Unclear	Unclear	Yes	Yes	No	No	Yes	Yes	Yes
Zacherl 2011	Yes	Unclear	Yes	No	Yes	Yes	Yes	Yes	Yes	Yes
Zhang 2015	Yes	Yes	Unclear	Yes	Yes	No	No	Yes	Yes	Yes
Ziewacz 2012	Yes	Yes	Unclear	Yes	Yes	Yes	No	Yes	Yes	Yes
