# Supplementary material for: Metastasectomy in Leiomyosarcoma: A Systematic Review and Pooled Survival Analysis
Source: Cancers (Basel). 2022 Jun 21;14(13):3055. doi: 10.3390/cancers14133055 (PMC9264936; doi:10.3390/cancers14133055)
Supplement: Supplementary file 1 [file cancers-14-03055-s001.zip › Supplementary Table S4.pdf]

Table S4. NOS for Cohort Studies

Criteria	Farid 2013	Van Cann 2018
Selection		
Representativeness of the exposed cohort	❋	❋
Selection of non exposed cohort	❋	❋
Ascertainment of exposure	❋	❋
Demonstration that outcome of interest was not present at start of study	❋	❋
Comparability		
Comparability of cohorts		❋
Outcome		
Assessment of outcome	❋	❋
Was follow-up long enough for outcomes to occur		❋
Adequacy of follow up of cohorts		❋
