# Supplementary material for: Metastasectomy in Leiomyosarcoma: A Systematic Review and Pooled Survival Analysis
Source: Cancers (Basel). 2022 Jun 21;14(13):3055. doi: 10.3390/cancers14133055 (PMC9264936; doi:10.3390/cancers14133055)
Supplement: Supplementary file 1 [file cancers-14-03055-s001.zip › Supplementary Table S5.pdf]

Table S5. Components of GRADE (Grading of Recommendation, Assessment, Development and Evaluation) to Assess the Certainty of Evidence

Grade Domain	Judgement	Concerns about certainty domains
Methodological limitations of the studies	All studies included were cohort or case series studies, resulting in confounding and selection bias due to lack of randomization.	Serious
Indirectness	The patients, interventions and comparators (when available) provided direct evidence of the clinical question at hand. All interventions included metastasectomy of individuals with at least 5 individuals with LMS	Not serious
Imprecision	The range of post-metastasectomy overall survival in studies varied greatly between studies included, with significant heterogeneity.	Serious
Inconsistency	The estimated post-metastasectomy overall survival in studies varied greatly between studies included, with significant heterogeneity.	Serious
Publication Bias	Comparative studies including patients who did not undergo metastasectomy or included sufficient details on alternative treatments were not widely available limiting the conclusion that can be drawn regarding the benefit of metastasectomy.	Serious
